# Zika infection and the eye

**Published:** 2020-03-30

**Authors:** Olivia A Zin, Andrea Zin

**Affiliations:** 1 Universidade Federal de São Paulo-UNIFESP São Paulo, Brazil.; 2 Instituto Fernandes Figueira-FIOCRUZ Rio de Janeiro, Brazil.

**If a woman becomes infected with the Zika virus while pregnant, the virus can cross the placenta and infect the baby, causing abnormalities of the eyes and the rest of the body. A detailed eye examination is essential.**

**Figure 1:**
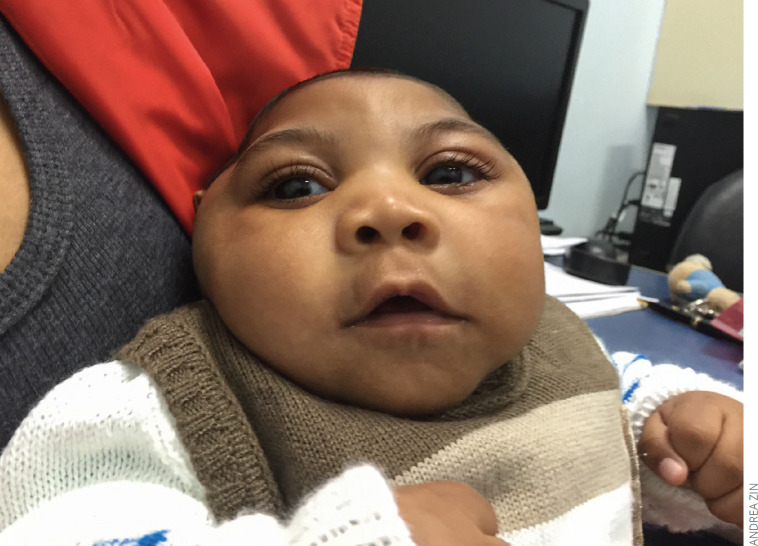
Child with congenital Zika syndrome and microcephaly

## Natural history and transmission

Zika virus (ZIKV) is a flavivirus (genus *Flavivirus*), first isolated in 1947 from a monkey in the Zika forest of Uganda. The virus is endemic in areas of Africa and Asia.

The virus can be spread by *Aedes* mosquitoes (which are active in the daytime) or via sexual contact, infected blood and trans-placental transmission in utero.^1^

**“Reports of Zika virus have increased recently, with cases being reported in new countries outside of Africa.”**

People carrying the virus can introduce Zika virus into new countries; however, Aedes mosquitoes are required to continue local transmission.

Reports of Zika virus have increased recently, with cases being reported in new countries outside of Africa. In early 2015, Zika infection was confirmed in Brazil, causing a large outbreak due to the lack of immunity in the population and the abundance of *Aedes aegypti* mosquitos.^2^

## Clinical features of acute infection

Most people infected with Zika virus are asymptomatic. Those who have symptoms may complain of mild fever, rash, painful joints and muscles, headache and conjunctivitis. Rarely, patients may develop acute uveitis and/or a maculopathy which is characterised by macular retinal pigment epithelium (RPE) changes with a grey annulus around the fovea on posterior segment examination and disruption of outer retinal and RPE integrity, as evidenced by an optical coherence tomography OCT scan.^3–5^

The Zika virus may be found in tears, therefore good hand hygiene to prevent person-to-person contamination is important.

## Congenital Zika syndrome

If a woman becomes infected with the Zika virus while pregnant, then the virus can cross the placenta and infect the unborn foetus. This results in congenital Zika syndrome (CZS), which consists of a spectrum of clinical manifestations observed in babies who have been exposed to Zika virus while in utero.

The main features are severe microcephaly with partially collapsed skull (Figure 1) and brain abnormalities (thin cerebral cortex and subcortical calcification). In the skeleton, there may be congenital contractures, arthrogryposis or clubfoot, with increased tone in the muscles. Hearing loss may also be present.

The eye abnormalities seen in CZS include retinal pigment mottling, chorioretinal atrophy, optic atrophy/hypoplasia and coloboma (Figures 2a and 2b). Other documented ocular abnormalities are microphthalmia, iris coloboma, lens subluxation, cataract, intraocular calcifications, and congenital glaucoma.^6–9^ Children with CZS are at an increased risk of blindness because of these serious, and often untreatable, ocular and neurological abnormalities (Figure 3).^10^

Exposure to the virus during the first three months of pregnancy appears to be associated with more severe manifestations, although CZS may occur after maternal exposure to the virus at any point during her pregnancy.

## Assessment for congenital Zika syndrome

All babies born to women who may have been exposed to Zika virus during pregnancy should undergo a full clinical evaluation by a paediatrician and examination of the eye (including dilated fundoscopy) by an ophthalmologist to determine if there is any evidence of CZS which will require management and follow-up.

**Figure 2a:**
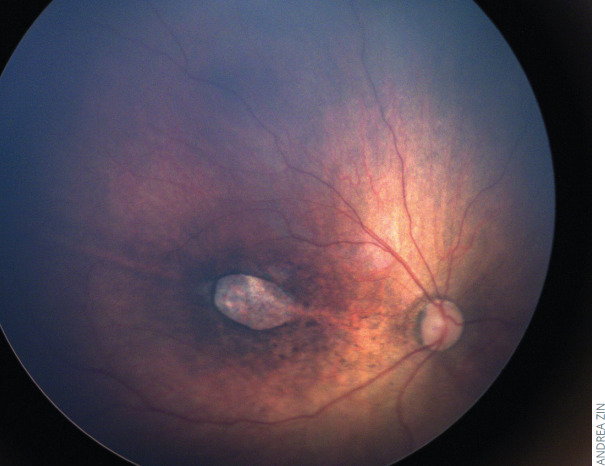
Chorioretinal atrophy and pigment mottling

**Figure 2b:**
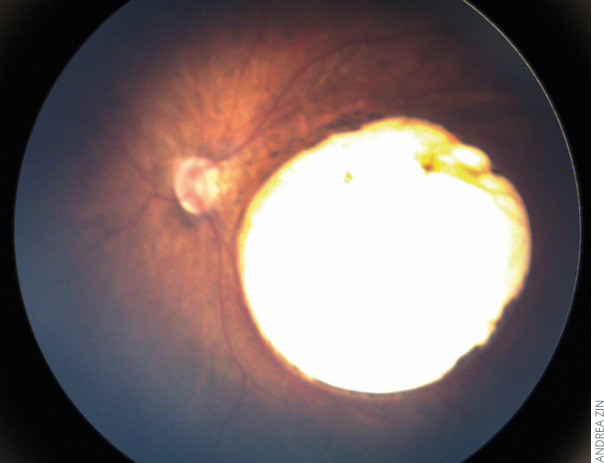
Optic nerve hypoplasia and macular coloboma

## Diagnostic tests to confirm clinical diagnosis

When the infection is acquired, most cases are asymptomatic and the symptoms, if present, are non-specific. If the patient is pregnant and concerned that she may have contracted Zika, then laboratory tests are required to confirm the infection.

Real-time polymerase chain reaction (RT-PCR) can identify the virus in blood samples 4 to 7 days after clinical onset. It is also possible to identify viral ribonucleic acid (RNA) in the urine up to 15 days after symptoms, even if the virus is no longer present in the bloodstream. Immunoglobulin (IgM) is increased between the 2nd and 12th week after clinical presentation; however, there may be cross-reactivity with other flaviviruses.

## Prevention and treatment

Zika virus infection can be prevented by avoiding mosquito bites (using mosquito repellent and mosquito nets; wearing long-sleeved shirts and trousers), particularly between sunrise and sunset, when Aedes mosquitos are most active.

The risk of sexual transmission of Zika virus is reduced by using condoms when staying in an endemic area and for 8 weeks after returning from this area. If symptoms of Zika infection have been noted, then condom use is recommended for 6 months after the infection.

Travellers returning from Zika-endemic areas should wait 28 days from their date of return before they can donate blood.

Currently, there is no specific antiviral treatment and no effective vaccine to prevent the infection.

**Figure 3:**
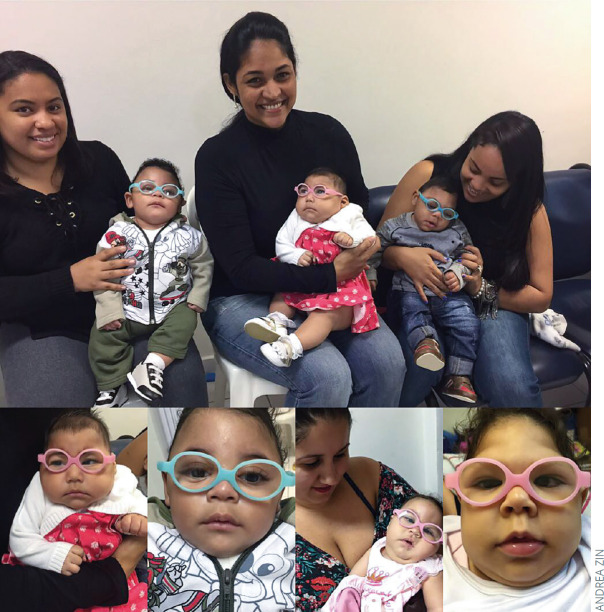
Children with congenital Zika syndrome wear magnifying glasses
